# Skin CD4^+^ Memory T Cells Play an Essential Role in Acquired Anti-Tick Immunity through Interleukin-3-Mediated Basophil Recruitment to Tick-Feeding Sites

**DOI:** 10.3389/fimmu.2017.01348

**Published:** 2017-10-16

**Authors:** Takuya Ohta, Soichiro Yoshikawa, Yuya Tabakawa, Kayoko Yamaji, Kenji Ishiwata, Hiroshi Shitara, Choji Taya, Masatsugu Oh-hora, Yohei Kawano, Kensuke Miyake, Yoshinori Yamanishi, Hiromichi Yonekawa, Naohiro Watanabe, Hirotaka Kanuka, Hajime Karasuyama

**Affiliations:** ^1^Department of Immune Regulation, Graduate School of Medical and Dental Sciences, Tokyo Medical and Dental University, Tokyo, Japan; ^2^Department of Tropical Medicine, The Jikei University School of Medicine, Tokyo, Japan; ^3^Laboratory for Transgenic Technology, Tokyo Metropolitan Institute of Medical Science, Tokyo, Japan; ^4^Division of Molecular Immunology, Medical Institute of Bioregulation, Kyushu University, Fukuoka, Japan; ^5^Bio-Oriented Technology Research Advancement Institution (BRAIN), Saitama, Japan

**Keywords:** tick infestation, protective immunity, basophils, CD4^+^ memory T cells, IL-3

## Abstract

Ticks, blood-sucking arthropods, serve as vectors for transmission of infectious diseases including Lyme borreliosis. After tick infestation, several animal species can develop resistance to subsequent infestations, reducing the risk of transmission. In a mouse model, basophils reportedly infiltrate tick-feeding sites during the second but not first infestation and play a crucial role in the expression of acquired tick resistance. However, the mechanism underlying basophil recruitment to the second tick-feeding site remains ill-defined. Here, we investigated cells and their products responsible for the basophil recruitment. Little or no basophil infiltration was detected in T-cell-deficient mice, and adoptive transfer of CD4^+^ but not CD8^+^ T cells reconstituted it. *Il3* gene expression was highly upregulated at the second tick-feeding site, and adoptive transfer of interleukin-3 (IL-3)-sufficient but not IL-3-deficient CD4^+^ T cells conferred the basophil infiltration on T-cell-deficient mice, indicating that the CD4^+^ T-cell-derived IL-3 is essential for the basophil recruitment. Notably, IL-3^+^ resident CD4^+^ memory T cells were detected even before the second infestation in previously uninfested skin distant from the first tick-feeding site. Taken together, IL-3 produced locally by skin CD4^+^ memory T cells appears to play a crucial role in basophil recruitment to the second tick-feeding site.

## Introduction

Ticks are blood-feeding ectoparasites that transmit a variety of pathogenic organisms, such as viruses, bacteria, protozoa, and helminths, many of which can cause various infectious disorders in human and animal hosts (1–3). During blood feeding and salivation, pathogenic microorganisms are delivered from infected ticks to hosts. Tick-borne diseases include viral encephalitis, sever fever with thrombocytopenia syndrome, Lyme disease, monocytic human ehrlichiosis, Rocky mountain spotted fever, and babesiosis (1–3). It has been shown that several animal species, including mice, guinea pigs, rabbits, and bovines, can develop resistance to tick feeding after single or repeated tick infestation, even though the expression of acquired resistance varies, depending the combination of different tick species and animal species/strains (4, 5). The resistance to tick feeding can result in reduced numbers and weights of engorged ticks or death of engorging ticks in re-infestation. From a clinical point of view, this acquired protective immunity to tick infestation is very important, because it reduces the chance of pathogen transmission that causes infectious diseases (6, 7).

Basophils are the least common granulocytes, accounting for less than 1% of peripheral blood leukocytes (8). Tick-feeding sites in guinea pigs with acquired resistance showed substantial infiltration of basophils (9–11). Basophils represent up to 70% of cellular infiltrates in the skin lesion, and basophil depletion with anti-basophil antiserum abolished tick resistance (12), illustrating a crucial role of basophils in acquired tick resistance. Later studies examined the contribution of basophils and mast cells to acquired tick resistance in mice infested with *Haemaphysalis longicornis* ticks (13–15). *H. longicornis* principally infests domestic animals such as cattle and is an important vector of pathogens causing babesiosis, Russian encephalitis, and Q fever in animals and humans (16). In mouse model of *H. longicornis* infestation, no apparent infiltration of basophils was histologically detected in the second tick-feeding site, although mice showed acquired tick resistance (13). Because mast cell-deficient mice failed to show tick resistance (13–15), it was suggested that mast cells, instead of basophils, play a crucial role in the acquired tick resistance in mice, unlike in guinea pigs. We previously revisited the contribution of basophils to acquired protective immunity against tick infestation in mice by using novel analytical tools, including basophil-specific anti-mMCP-8 antibody and engineered mice deficient for only basophils, *Mcpt8*^DTR^ mice (17). Whereas Giemsa staining of skin sections failed to identify basophil infiltration at *H. longicornis*-feeding sites, in accordance with earlier reports (13), immunohistochemical analysis with anti-mMCP-8 antibody revealed the basophil accumulation at the second but not first tick-feeding site (17). Importantly, basophil depletion just before the second infestation almost completely abolished the acquired tick resistance (17). Consistent with the previous study, mice deficient for mast cells failed to show acquired tick resistance (17). Thus, both basophils and mast cells are essential for tick resistance in mice. We further demonstrated that IgFc receptors expressed on basophils but not mast cells are essential for the expression of acquired tick resistance (17). Our study, together with the previous study in guinea pig, illustrated that basophils play a pivotal and non-redundant role in antibody-mediated acquired resistance to tick infestation. Nevertheless, it remains totally unknown how basophils are recruited to the second tick-feeding site distant from the first infestation site, leading to tick resistance.

In the present study, we investigated cells and their products responsible for the recruitment of basophils that contribute to acquired tick resistance. We here demonstrate that skin CD4^+^ memory T cells play an essential role in acquired protective immunity against tick infestations through the production of interleukin-3 (IL-3) that in turn induces basophil recruitment to the tick re-infestation site.

## Materials and Methods

### Mice

C57BL/6J mice were purchased from Japan SLC. *Mcpt8*^GFP^ (18), *Rag2*^−/−^ (19), *Il3*^−/−^ (20), and *Fcer1g*^−^*^/^*^−^ (21) mice on the C57BL/6J background were described previously. OT-II Tg/*Rag2*^−/−^ mice (22) were kindly provided by Dr. Francis R. Carbone. *Tcr*α^−/−^ mice (23) were obtained from the Jackson laboratory. All mice were maintained under specific pathogen-free conditions in our animal facilities in accordance with the guidelines of the Tokyo Medical and Dental University for animal care, and all animal studies were approved by the Institution Animal Care and Use Committee of Tokyo Medical and Dental University (permit number: 0170087A).

### Tick Infestation

*Haemaphysalis longicornis* of the laboratory-reared strain were used to infest mice. Tick infestation was performed as described previously (17). To examine the tick resistance, mice were infested twice at an interval of 14 days with 40 larvae each time at two different locations, the left flank for the first infestation and the right flank for the second infestation. Most of ticks were detached from the host by day 5 of each infestation.

### Antibodies and Reagents

Following reagents were purchased from Biolegend: allophycocyanin (APC)-conjugated anti-CD200R3 (Ba13), anti-CD44 (IM7), Rat IgG2b, and κ (RTK4530); phycoerythrin (PE)-conjugated anti-IL-3 (MP2-8F8), anti-CD63 (NVG-2), Rat IgG1, κ (RTK2071), Rat IgG2a, and κ (RTK2758); fluorescein isothiocyanate (FITC)-conjugated anti-CD49b (HMα2) and anti-CD62L (MEL-14); PE/Cy7-conjugated anti-CD69 (H1.2F3); APC/Cy7-conjugated anti-CD3ε (145-2C11); Pacific Blue™-conjugated anti-CD117 (2B8); brilliant violet 421™-conjugated anti-CD4 (RM4.5); and biotin-conjugated anti-CD4 (GK1.5), anti-CD8a (53-6.7), and recombinant IL-3. Isoflurane and normal rat serum were obtained from WAKO. Anti-CD16/32 mAb (2.4G2) was prepared in our laboratory.

### Flow Cytometry

Single-cell suspensions were prepared by treating the flank skin with 125 U/ml collagenase (Wako) at 37°C for 2 h. Isolated cells were preincubated with anti-CD16/32 for 30 min to block non-specific binding of antibodies, subsequently stained with indicated combination of antibodies, and then analyzed with FACSCanto II (BD Biosciences) and FlowJo (Tree star). Each cell lineage was identified as follows: basophils (c-kit^−^CD49b^+^CD200R3^+^), activated basophils (c-kit^−^CD49b^+^CD200R3^+^CD63^+^), mast cells (c-kit^+^CD49b^+^CD200R3^+^), naive T cells (CD3^+^CD62L^+^CD44^−^), T_CM_ cells (CD3^+^CD62L^+^CD44^+^), T_EM_ cells (CD3^+^CD62L^−^CD44^+^CD69^−^), and T_RM_ cells (CD3^+^CD62L^+^CD44^+^CD69^+^). For cytoplasmic staining of cytokines, cells were stimulated for 6 h with phorbol 12-myristate 13-acetate (PMA 0.1 µg/ml; Sigma-Aldrich) plus ionomycin (1 µM; Sigma-Aldrich) in the presence of monensin (BD GolgiStop; BD Biosciences) for the last 2 h. Subsequently, cells were stained with CD3ε, CD4, CD44, CD62L, and CD69 to label surface markers. Cells were then treated with BD Cytofix/Cytoperm Fixation and Permeabilization Solution (BD Biosciences) and stained with anti-IL-3.

### RNA Preparation and Real-time Quantitative Reverse Transcription PCR

Total mRNAs from skin tissues were prepared by using RNeasy Mini Kit (Qiagen). cDNAs were synthesized using reverse transcription using ReverTra Ace (TOYOBO) and oligo-dT primers. Quantitative PCR was performed in StepOnePlus™ Real-Time PCR system (Applied Biosystems) using a Fast SYBR Green Master Mix (Applied Biosystems) and the following primer sets:

*Gapdh* (sense-AGGTCGGTGTGAACGGATTTG and antisense-TGTAGACCATGTAGTTGAGGTCA),
*Ifng* (sense-ACAGCAAGGCGAAAAAGGATG and antisense-TGGTGGACCACTCGGATGA),
*Il3* (sense-GGGATACCCACCGTTTAACCA and antisense-AGGTTTACTCTCCGAAAGCTCTT),
*Il4* (sense-ATCATCGGCATTTTGAACGAGG and antisense-TGCAGCTCCATGAGAACACTA),
*Il17a* (sense-TGTGAAGGTCAACCTCAAAGTC and antisense-AGGGATATCTATCAGGGTCTTCATT).
*Il5* (sense-CTCTGTTGACAAGCAATGAGACG and antisense-TCTTCAGTATGTCTAGCCCCTG),
*Il13* (sense-GCAACATCACACAAGACCAGA and antisense-GTCAGGGAATCCAGGGCTAC).

The expression of each cytokine gene was normalized by using *Gapdh* expression as a reference.

### Adoptive Transfer of T Cells

Splenic cells isolated from wild-type (WT), *Il3*^−/−^, or OT-II Tg/*Rag2*^−/−^ mice were incubated with biotinylated-anti-CD4 or CD8 antibody at 4°C for 30 min, followed by positive isolation of CD4^+^ or CD8^+^ T cells using BD IMag™ (BD Biosciences). T cells (2 × 10^6^ cells per recipient) were intravenously transferred into *Rag2*^−/−^ mice 24 h before the first infestation.

### Continuous Administration of Recombinant IL-3

*Rag2*^−/−^ mice were treated daily with subcutaneous administration of recombinant IL-3 (100 ng/injection/day) in the right flank and control PBS in the left flank for 10 days from day −7 to day 2, and on day 0, mice were primarily infested with ticks at the administration sites of both flanks.

### *In Vivo* Fluorescent Imaging

Mice were anesthetized with 1.5% isoflurane gas and covered with a heating blanket to keep their body temperature at 37°C. Intravital images of the flank skin were captured with an inverted laser scanning microscope (A1R^+^; Nikon) equipped with CFI Plan Apochromat λ ×10 or ×20 objective lens or fluorescence stereo microscopes (Leica). NIS elements, Volocity, and Imaris software were used for acquisition and analysis of images.

### Statistical Analysis

Statistical analysis was performed with unpaired Student’s *t*-test. *P* value <0.05 was considered statistically significant.

## Results

### Basophils Accumulate at Tick-Feeding Sites at the Early Phase of the Second Tick Infestation in an Antibody-Independent Manner

**Figure 1:**
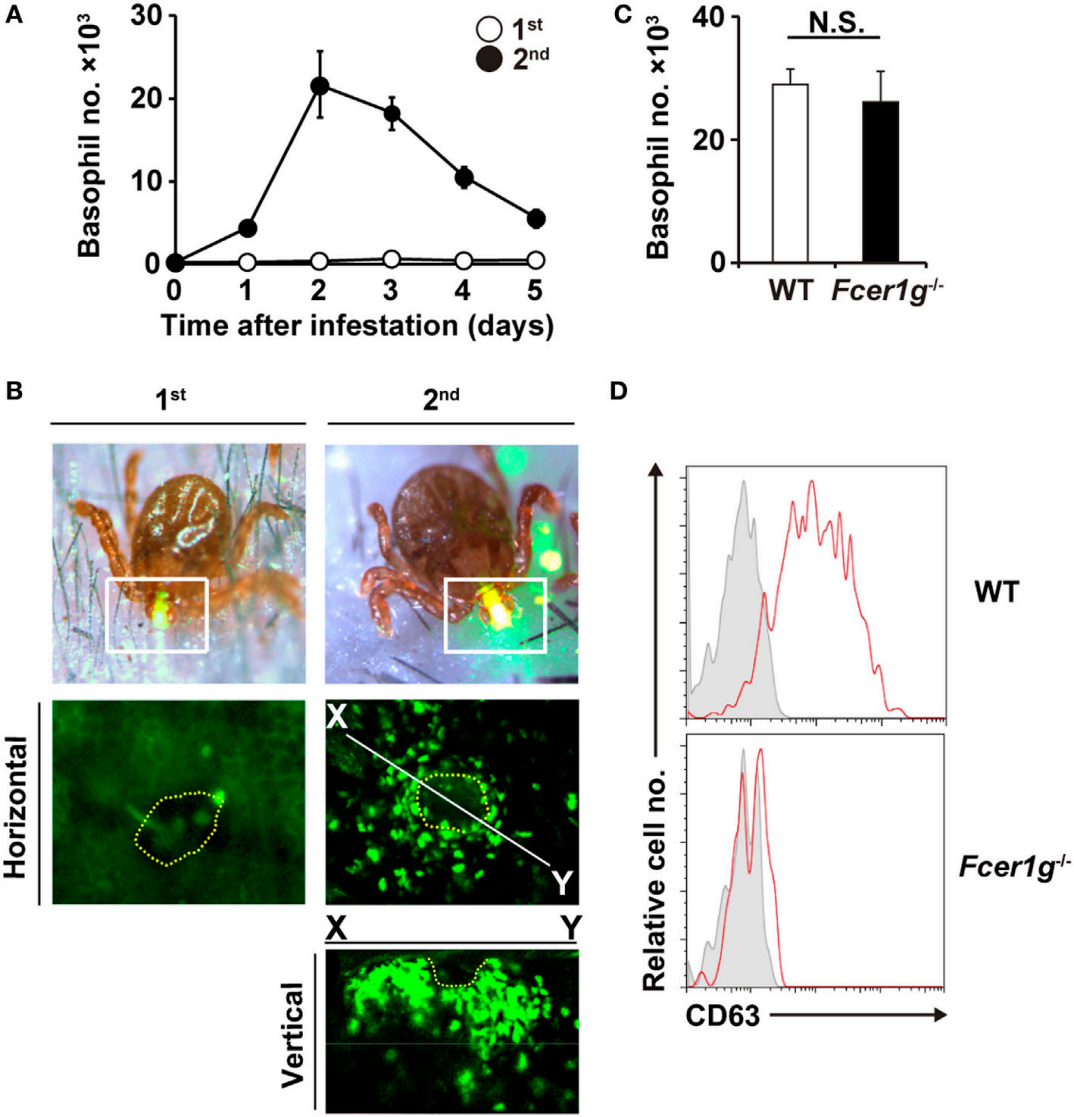
Basophils accumulate at tick-feeding sites at the early phase of the second tick infestation in an antibody-independent manner. **(A)** C57BL/6 mice were infested with larval ticks once or twice at an interval of 14 days. The number of basophils accumulating at tick-feeding sites (mean ± SEM, *n* = 3 each) was counted at the indicated time points during the first (white circles) and second (black circles) infestations. **(B)** *Mcpt8*^GFP^ mice were infested with ticks one or twice and subjected to intravital imaging analysis of green basophils at tick-feeding sites on day 2 of the first or second infestation. Dashed yellow lines indicate the places where tick mouthparts were inserted into the skin. **(C,D)** Wild-type (WT) or *Fcer1g*^−/−^ mice were infested twice with ticks. The number of basophils at tick-feeding sites [**(C)**, mean ± SEM, *n* = 3 each] and their surface expression of CD63 **(D)** were examined on day 2 of the second infestation. Shaded histograms indicate staining with isotype-matched control antibody. All the data shown are representative of three independent experiments. N.S., not significant.

We first examined time course of basophil accumulation at tick-feeding sites during the first and second infestations (Figure 1A). In accordance with our previous study (17), no apparent infiltration of basophils was detected during the first infestation. In contrast, during the second infestation at the skin distant from the first tick infestation site, basophils infiltrated and accumulated in the skin lesion, with a peak of cell number on day 2 or 3 post-infestation (Figure 1A). The decrease of basophil number at the second tick-feeding site after day 3 appeared to coincide with the reduced number of ticks staying attached to the skin due to drop-off of engorged ticks (Figure S1 in Supplementary Material). Intravital imaging, using *Mcpt8*^GFP^ mice in that only basophils express GFP (18), revealed that green basophils migrated toward and surrounded a tick mouthpart during the second infestation (Figure 1B; Movie S1 in Supplementary Material). In contrast to substantial infiltration of basophils at the second tick-feeding site, the number of mast cells at tick-feeding sites moderately increased during both the first and second infestation (Figure S2 in Supplementary Material).

We previously reported that basophil depletion prior to the second infestation completely abolished the resistance against tick feeding (17), illustrating the pivotal role of basophils in acquired tick resistance (17). Mice deficient for B cells (μMT mice) failed to show the acquired tick resistance in the second infestation (17). This was also the case in mice deficient for FcεRI-γ chain (*Fcer1g*^−^*^/^*^−^ mice) that lack high-affinity IgE receptor and activating IgG receptors (17). These data demonstrated that antibodies are involved in the manifestation of tick resistance. Of note, basophil accumulation at the second tick-feeding site was normally detected in μMT mice (17), suggesting antibody-independent basophil recruitment. In the present study, we first examined this assumption by analyzing *Fcer1g*^−^*^/^*^−^ mice. These mice showed basophil accumulation at the second tick-feeding site to an extent comparable to that detected in WT mice (Figure 1C), further supporting the dispensable role of antibody in basophil recruitment. Importantly, increased expression of CD63 was detected on the surface of basophils accumulating at the second tick-feeding site of WT but not *Fcer1g*^−^*^/^*^−^ mice (Figure 1D). These results suggest that FcεRI-γ-deficient basophils can be recruited to the tick-feeding site but not activated to inhibit tick feeding due to the lack of stimulation with tick antigens and specific antibodies.

### CD4^+^ T Cells Are Essential for Basophil Accumulation at the Second Tick-Feeding Site

**Figure 2:**
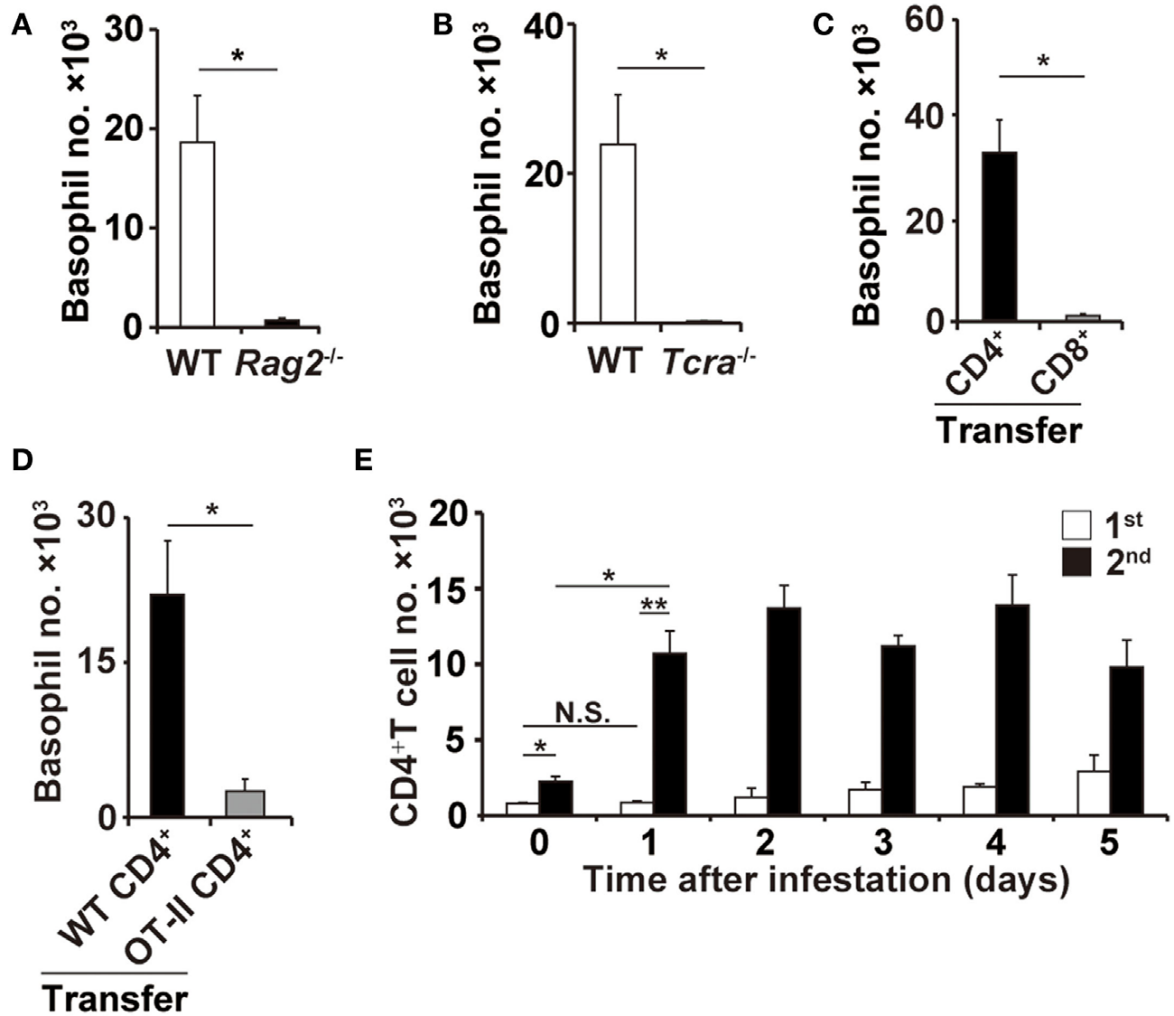
CD4^+^ T cells are essential for basophil accumulation at the second tick-feeding site. **(A,B)** Wild-type (WT), *Rag2*^−/−^, or *Tcra*^−/−^ mice were infested twice with ticks. The number of basophils (mean ± SEM, *n* = 3 each) at the second tick-feeding site in each mouse strain was examined on day 2 of infestation. **(C)** CD4^+^ or CD8^+^ T cells were prepared from the spleen of WT mice and adoptively transferred to *Rag2*^−/−^ mice. Recipient mice were infested twice with ticks, and the basophil number at the second tick-feeding site (mean ± SEM, *n* = 4 each) was examined as in **(A,B)**. **(D)** CD4^+^ T cells isolated from WT or OT-II Tg*/Rag2*^−/−^ mice were adoptively transferred to *Rag2*^−/−^ mice, and the basophil number (mean ± SEM, *n* = 4 each) at the second tick-feeding site of recipient mice was examined as in **(C)**. **(E)** WT mice were infested once or twice with ticks, and the number of CD4^+^ T cells at the first (white bars) and second (black bars) tick-feeding sites was examined (mean ± SEM, *n* = 3 each). All the data shown are representative of three independent experiments. N.S., not significant; *p < 0.05; **p < 0.01.

In contrast to μMT and *Fcer1g*^−^*^/^*^−^ mice, *Rag2*^−/−^ mice deficient for both T and B cells showed little or no accumulation of basophils at the second tick-feeding site (Figure 2A), suggesting the contribution of T cells to basophil recruitment. Indeed, the basophil accumulation was not detected in *Tcra*^−/−^ mice deficient for T-cell receptor α chain (Figure 2B). Adoptive transfer of CD4^+^ T cells but not CD8^+^ T cells isolated from WT mice conferred the basophil accumulation at the second tick-feeding site on *Rag2*^−/−^ mice (Figure 2C), indicating the important role of CD4^+^ T cells in the basophil accumulation. Such basophil accumulation was not detected at the first tick-feeding site unlike at the second tick-feeding site (Figure S3 in Supplementary Material). Of note, adoptive transfer of ovalbumin-specific CD4^+^ T cells isolated from OT-II transgenic mice failed to reconstitute the basophil accumulation at the second tick-feeding site in *Rag2*^−/−^ mice (Figure 2D), implying the importance of antigen specificity of CD4^+^ T cells in promoting basophil recruitment. Adoptive transfer of CD4^+^ T cells isolated from previously tick-infested WT mice but not from uninfested mice could confer basophil accumulation at the tick-feeding site even in the first infestation on *Rag2*^−/−^ mice (Figure S4 in Supplementary Material), suggesting CD4^+^ memory T cells, most likely carrying specificity to tick saliva antigens, may be crucial for triggering basophil recruitment to the tick-feeding site. In accordance with this assumption, the number of CD4^+^ T cells in the tick-feeding site increased as early as on day 1 of the second but not first tick infestation (Figure 2E).

### IL-3 Is Necessary and Sufficient for Basophil Accumulation at the Tick-Feeding Site

**Figure 3:**
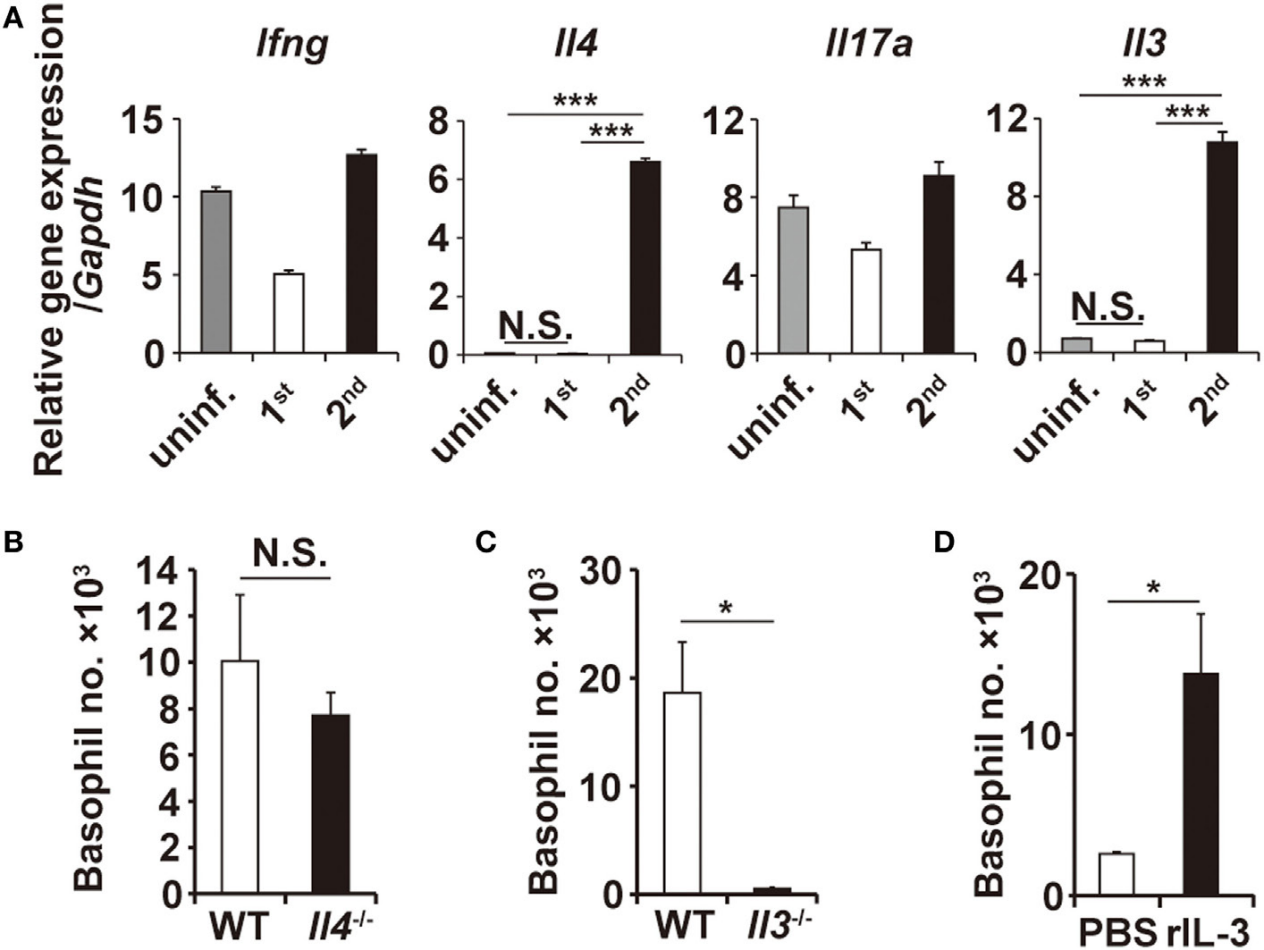
Interleukin-3 (IL-3) is necessary and sufficient for basophil accumulation at the tick-feeding site. **(A)** The transcriptional expression of indicated genes at uninfested skin, first or second tick-feeding site on day 2 of infestation is shown (mean ± SEM, *n* = 3 each). **(B,C)** Wild-type, *Il4*^−/−^, or *Il3*^−/−^ mice were infested twice with ticks, and the number of basophils (mean ± SEM, *n* = 4 each) at the second tick-feeding site in each mouse strain was examined on day 2 of infestation. **(D)** *Rag2*^−/−^ mice were treated daily with subcutaneous administration of recombinant IL-3 (100 ng/injection/day) in the right flank and control PBS in the left flank for 10 days from day −7 to day 2, and on day 0, mice were primarily infested with ticks at the administration sites of both flanks. The number of basophils (mean ± SEM, *n* = 4 each) at the first tick-feeding site of each side of flank was examined on day 2 of infestation. All the data shown are representative of three independent experiments. N.S., not significant; *p < 0.05; ***p < 0.001.

T-cell-derived cytokines reportedly contribute to the recruitment of granulocytes (24–26). RT-PCR analysis revealed that the transcription of *Il3* and *Il4* genes, among the genes analyzed, was highly upregulated at the tick-feeding site during the second but not first infestation, compared to that in uninfested skin (Figure 3A; Figure S5 in Supplementary Material). To examine the possible contribution of IL-3 and IL-4 to the skin infiltration of basophils, mice deficient for either IL-3 or IL-4 were infested with ticks twice. Basophil accumulation at the second tick-feeding site was detected in IL-4-deficient mice to an extent comparable to that observed in WT mice (Figure 3B). By contrast, little or no accumulation was detected in IL-3-deficient mice (Figure 3C), indicating the essential role of IL-3 for the basophil accumulation. We next explored whether local production of IL-3 in the tick-feeding site is sufficient for basophil recruitment and accumulation. To this end, *Rag2*^−/−^ mice were treated daily with subcutaneous administration of recombinant IL-3 in the right flank and control PBS in the left flank for 10 days from day −7 to day 2, and on day 0, mice were primarily infested with ticks at the administration sites of both flanks. Even in the absence of T cells, significant accumulation of basophils was detected at the IL-3-treated but not PBS-treated flank during the first infestation (Figure 3D; Figure S6A in Supplementary Material). These results strongly suggested that IL-3 produced locally at the tick-feeding site is necessary and sufficient for basophil recruitment and accumulation, even though we cannot formally exclude the possibility that other factors may modulate them.

### IL-3 Produced by CD4^+^ T Cells Is Responsible for Basophil Accumulation at the Second Tick-Feeding Site

**Figure 4:**
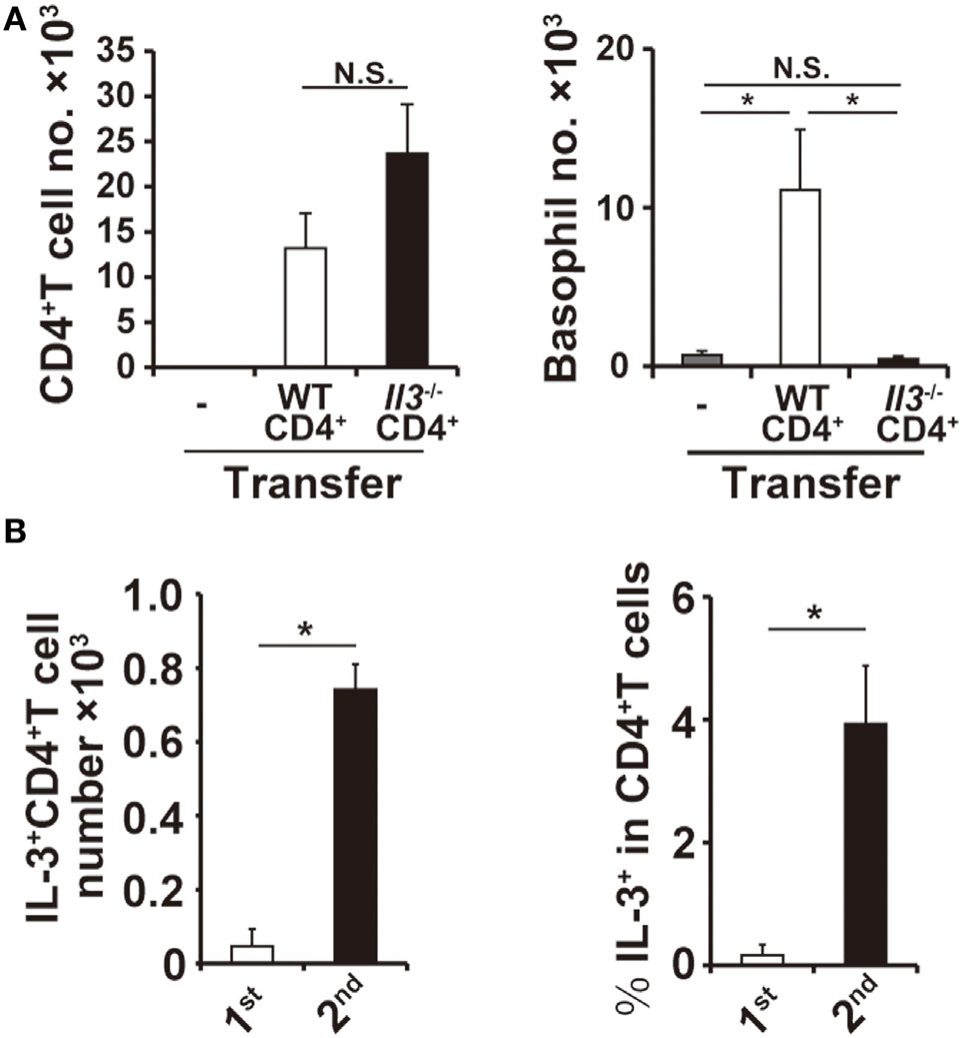
Interleukin-3 (IL-3) produced by CD4^+^ T cells is responsible for basophil accumulation at the second tick-feeding site. **(A)** Splenic CD4^+^ T cells isolated from wild-type (WT) or *Il3*^−/−^ mice were adoptively transferred into *Rag2*^−/−^ mice, and recipient mice were infested twice with ticks. The number of CD4^+^ T cells (left panel) and basophils (right panel) at the second tick-feeding site was examined on day 2 of infestation (mean ± SEM, *n* = 4 each). **(B)** WT mice were infested twice with ticks, and the number of IL-3^+^CD4^+^ T cells at the first and second tick-feeding sites (left panel) and the frequency of IL-3^+^ T cells among CD4^+^ T cells at the first and second tick-feeding sites (right panel) were determined (mean ± SEM, *n* = 3 each). All the data shown are representative of three independent experiments. N.S., not significant; *p < 0.05.

The findings that both CD4^+^ T cells and IL-3 are important for the basophil accumulation prompted us to examine the possibility that IL-3 produced by CD4^+^ T cells is responsible for the basophil accumulation at the second tick-feeding site. Adoptive transfer of CD4^+^ T cells from IL-3-deficient mice, unlike those from WT mice, failed to confer the basophil accumulation at the second tick-feeding site on *Rag2*^−/−^ mice, even though the number of IL-3-deficient CD4^+^ T cells accumulating at the tick-feeding site was comparable to that of WT CD4^+^ T cells (Figure 4A; Figure S6B in Supplementary Material). The *ex vivo* stimulation of CD4^+^ T cells isolated from the tick-feeding site, followed by intracellular staining for IL-3, revealed that the number of CD4^+^ T cells with IL-3-producing capacity (hereafter referred to as IL-3^+^CD4^+^ T cells) significantly increased at the tick-feeding site during the second infestation compared to that during the first infestation (Figure 4B, left panel), in parallel with the extent of basophil accumulation at the tick-feeding site. The frequency of IL-3-producing cells among CD4^+^ T cells at the second tick-feeding site on day 2 of infestation was 1–5% (Figure 4B, right panel). All these results suggested that a fraction of CD4^+^ T cells at the second tick-feeding site can produce IL-3 that in turn induces basophil infiltration into the skin lesion.

### IL-3^+^ CD4^+^ T Cells at the Second Tick-Feeding Site Show a Memory Phenotype, Including Resident and Effector Memory T Cells

**Figure 5:**
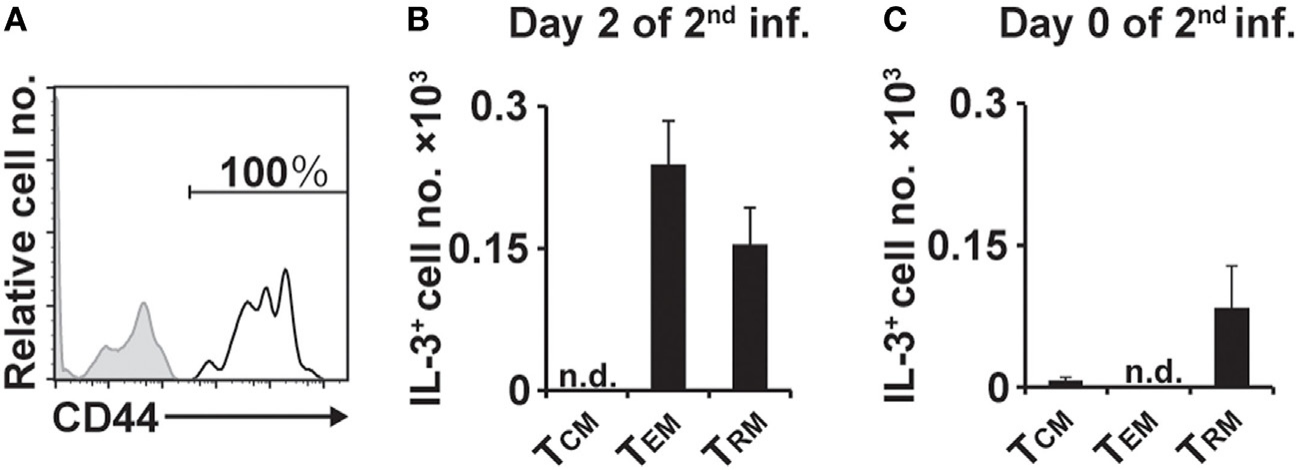
Interleukin-3 (IL-3)^+^CD4^+^ T cells at the second tick-feeding site show memory phenotypes, including T_RM_ and T_EM_ cells. **(A)** The expression of CD44 (open histogram) on IL-3^+^CD4^+^ T cells accumulating at the second tick-feeding site of wild-type mice is shown. A shaded histogram indicates staining with isotype-matched control antibody. **(B,C)** The number of IL-3^+^CD4^+^ T cells showing the phenotype of T_CM_ (CD3^+^CD62L^+^CD44^+^), T_EM_ (CD3^+^CD62L^−^CD44^+^CD69^−^), or T_RM_ (CD44^+^CD62L^−^CD69^+^) on day 2 **(B)** and on day 0 **(C)** of the second infestation is shown (mean ± SEM, *n* = 3 each). All the data shown are representative of three independent experiments. n.d., not detected.

We next examined the surface phenotype of IL-3^+^CD4^+^ T cells at the second tick-feeding site. Virtually all of them present on day 2 of the second infestation expressed CD44 (Figure 5A), suggesting that they are memory T cells (27). CD4^+^ memory T cells include central memory (T_CM_), effector memory (T_EM_), and resident memory (T_RM_) cells (28–32). Among IL-3^+^CD44^+^CD4^+^ T cells present at the second tick-feeding site on day 2 of infestation, approximately 60 and 40% showed the surface phenotype of T_EM_ and T_RM_, respectively, and T cells with the T_CM_ phenotype was barely detected (Figure 5B). Notably, IL-3^+^CD4^+^ T cells with the T_RM_ but not T_EM_ phenotype were detected on day 0 of the second infestation, namely 14 days after the initiation of the first infestation and immediately before the second infestation (Figure 5C). Such IL-3^+^CD4^+^ T cells with the T_RM_ phenotype were hardly detected in the skin of naive mice without any experience of tick infestation (data not shown). When CD4^+^ T cells isolated from uninfested WT mice were adoptively transferred into *Rag2*^−/−^ mice, some of them were detected as T_RM_ cells at previously uninfested skin on day 14 of the first infestation (Figure S7 in Supplementary Material). These results suggested that after the first infestation, IL-3^+^CD4^+^ T_RM_ cells, most likely carrying specificity to tick antigens, were generated and widely distributed to the skin distant from the first tick-feeding site.

## Discussion

Basophils have been shown to infiltrate and accumulate at tick-feeding sites during re-infestation but not the first infestation and are critically involved in the manifestation of acquired resistance to tick feeding (12, 17). However, the mechanism underlying basophil recruitment to the second tick-feeding site, far from the first infestation site, remained ill-defined. In the present study, we identified cells and their product that are responsible for the basophil recruitment. Adoptive transfer experiments revealed that IL-3 produced by CD4^+^ T cells is essential for the basophil recruitment to the second tick-feeding site. IL-3^+^CD4^+^ T cells were readily detected at the second but not first tick-feeding site, and virtually all of them showed the memory phonotype. Importantly, IL-3^+^CD4^+^ T_RM_ cells were detected in previously uninfested skin, distant from the first tick-feeding site, of previously infested mice. Thus, skin CD4^+^ memory T cells play a pivotal role in acquired tick resistance through local production of IL-3, most likely in response to stimulation with tick antigens, that in turn promotes skin infiltration of basophils necessary for the manifestation of resistance to tick feeding.

T_RM_ cells are a recently described subset of memory T cells that stay long term in peripheral tissues, in contrast to circulating memory T-cell subsets including T_CM_ and T_EM_ cells (29–32). T_RM_ cells are derived from precursor cells, which entered tissues during the effector phase of primary response and stayed within the tissues. Therefore, they can respond readily to pathogen challenge at the sites independently of recruitment of blood-circulating T cells. T_RM_ cells have been extensively characterized among the CD8^+^ T-cell subset that plays a crucial role in protection from viral infections. CD8^+^ T_RM_ cells have been shown to directly control local infection, including killing of infected cells, and indirectly modify the tissue environment to promote inflammation (29–32). Recent studies reported the presence of CD4^+^ T_RM_ cells in peripheral tissues such as the lung, genital tract, and skin (33–37), even though less is known about their functions compared to those of CD8^+^ T_RM_ cells. Skin-resident, IFNγ-producing CD4^+^ memory T cells protect against *Leishmania major* re-infection by recruiting circulating T cells and inflammatory monocytes to the site of re-infection, leading to efficient control of parasitic infections (38, 39). In the present study, we demonstrated for the first time that IL-3-producing CD4^+^ memory T cells, including T_RM_ cells, play an essential role in the basophil recruitment to the site of tick re-infestation and, thereby, contribute to the acquired protective immunity against tick infestation. Even before the second tick infestation, IL-3^+^CD4^+^ T_RM_ cells could be detected in previously uninfested skin far from the first tick-feeding site, suggesting that CD4^+^ effector T cells generated during the first infestation migrated into the skin throughout the body, and some of them were retained as T_RM_ cells in the skin. The number of IL-3^+^CD4^+^ T_RM_ cells at the second tick-feeding site increased up to two-fold during the first 2 days of re-infestation (Figures 5B,C). IL-3^+^CD4^+^ T_EM_ cells were barely detected in the skin on day 0 of the second infestation but rapidly accumulated by day 2 (Figures 5B,C), indicating that circulating CD4^+^ T_EM_ cells were recruited to the second tick-feeding site. Although it remains to be investigated whether CD4^+^ T_RM_ cells contribute to the recruitment of circulating CD4^+^ T_EM_ cells as reported in the *L. major* re-infection (38), IL-3^+^CD4^+^ T_EM_ cells recruited to the second tick-feeding site, in addition to pre-existing IL-3^+^CD4^+^ T_RM_ cells, appear to further promote the basophil recruitment to the skin. We showed here the importance of IL-3 produced by skin CD4^+^ memory T cells in acquired anti-tick immunity while IFNγ produced by skin CD4^+^ memory T cells plays an important role in acquired immunity against *Leishmania* infection (38). This suggests that skin CD4^+^ memory T cells may utilize distinct cytokines to combat with different species of parasites.

A previous study reported that basophils are transiently recruited to draining lymph nodes when mice are primarily infected with helminth *Nippostrongylus brasiliensis* (40). Similarly, we detected basophil recruitment to draining lymph nodes during the first tick infestation (17). In contrast, basophil recruitment to *N. brasiliensis*-infected skin lesions was detected during the second but not first infections (41), as observed in the tick-infested skin. Taking together, the basophil recruitment to draining lymph nodes occurs in the primary immune response to the parasitic infections whereas that to the skin takes place only in recall response. It has been shown that IL-3 is required for basophil recruitment to draining lymph nodes in primary *N. brasiliensis* infection and CD4^+^ T cells are the major producer of IL-3 (40). Therefore, the requirement of IL-3 produced by CD4^+^ T cells appears to be common to the basophil recruitment to draining lymph nodes and skin in the parasitic infections, in spite of the difference in the timing of recruitment. Of note, IL-3^+^CD4^+^ T cells were detected in draining lymph nodes during the first tick infestation (data not shown), whereas they were detectable at the skin during the second but not first infestation, suggesting the importance of CD4^+^ memory T cells in the skin but not in lymph nodes. Indeed, IL-3^+^CD4^+^ T cells present in draining lymph nodes during the first tick infestation expressed low levels of CD44 (data not shown) in contrast to IL-3^+^CD4^+^ T cells in the second tick-feeding site. Such IL-3^+^CD4^+^-naive T cells were barely detected in the skin, unlikely in draining lymph nodes, during the first tick infestation (data not shown). Taken together, the phenotypic difference of IL-3^+^CD4^+^ T cells, naive versus memory, as well as their distinct localization appears to account for the different timing of basophil recruitment in the skin and lymph nodes. Of note, another study showed that inhalation of *Aspergillus fumigatus* extract induced prompt increase in basophil numbers in the lung in a manner independent of T cells and IL-3 (42), suggesting that T-cell-derived IL-3 is not always the absolute requirement for basophil recruitment to peripheral tissues.

The exact mechanism by which IL-3 promotes basophil recruitment to the skin in tick re-infestation remains to be investigated. Previous studies demonstrated that endothelial cells express IL-3 receptor, and IL-3 can activate them to promote rolling and adhesion of basophils on endothelium in an adhesion molecule- and chemokine-dependent manner (43, 44). IL-3 also acts on basophils to enhance their adhesion onto endothelium (45). Therefore, IL-3 appears to induce tissue infiltration of basophils at least in part by promoting basophil adhesion to endothelium, leading to transendothelial migration. Our data suggested that IL-3 may also contribute to the accumulation of neutrophils at the tick-feeding site (Figure S6 in Supplementary Material).

In conclusion, we have defined the mechanism by which basophils infiltrate the skin of tick re-infestation. Skin CD4^+^ memory T cells play an essential role in acquired protective immunity to tick infestation through the production of IL-3 that in turn induces basophil recruitment to the site of tick re-infestation. This illustrates the excellent collaboration of T cells and basophils, belonging to the adaptive and innate immune systems, respectively, for protection against ticks that cause serious infectious diseases.

## Ethics Statement

This study was carried out in accordance with the guidelines of the Tokyo Medical and Dental University for animal care. The protocol was approved by the Institution Animal Care and Use Committee of Tokyo Medical and Dental University.

## Author Contributions

TO, SY, MO, YK, KM, YY, HiK, NW, and HaK designed the research. TO, SY, and YT performed experiments and analyzed data. KY, KI, and YT prepared ticks suitable for infestation. SY, HS, CT, and HY generated *Mcpt8*^GFP^ mice. HaK and SY supervised the work. TO, SY, and HaK wrote the manuscript. All authors provided critical review of the manuscript.

## Conflict of Interest Statement

The authors declare that the research was conducted in the absence of any commercial or financial relationships that could be construed as a potential conflict of interest.

## Supplementary Material

Movie S1 Basophils accumulate around a tick mouthpart at the second tick-feeding site. *Mcpt8*^GFP^ mice were infested twice with ticks and subjected to intravital imaging analysis of green basophils at tick-feeding sites on day 2 of the second infestation as in Figure 1B. Images were taken at 2 min intervals for 1.5 h.

## Abbreviations

IL-3, interleukin-3; T_RM_, tissue-resident memory T; T_EM_, effector memory T; T_CM_, central memory T.
