# Supplementary material for: Skin CD4+ Memory T Cells Play an Essential Role in Acquired Anti-Tick Immunity through Interleukin-3-Mediated Basophil Recruitment to Tick-Feeding Sites
Source: Front Immunol. 2017 Oct 16;8:1348. doi: 10.3389/fimmu.2017.01348 (PMC5650685; doi:10.3389/fimmu.2017.01348)
Supplement: Supplementary file 2 [file image_1.pdf]

Supplementary material

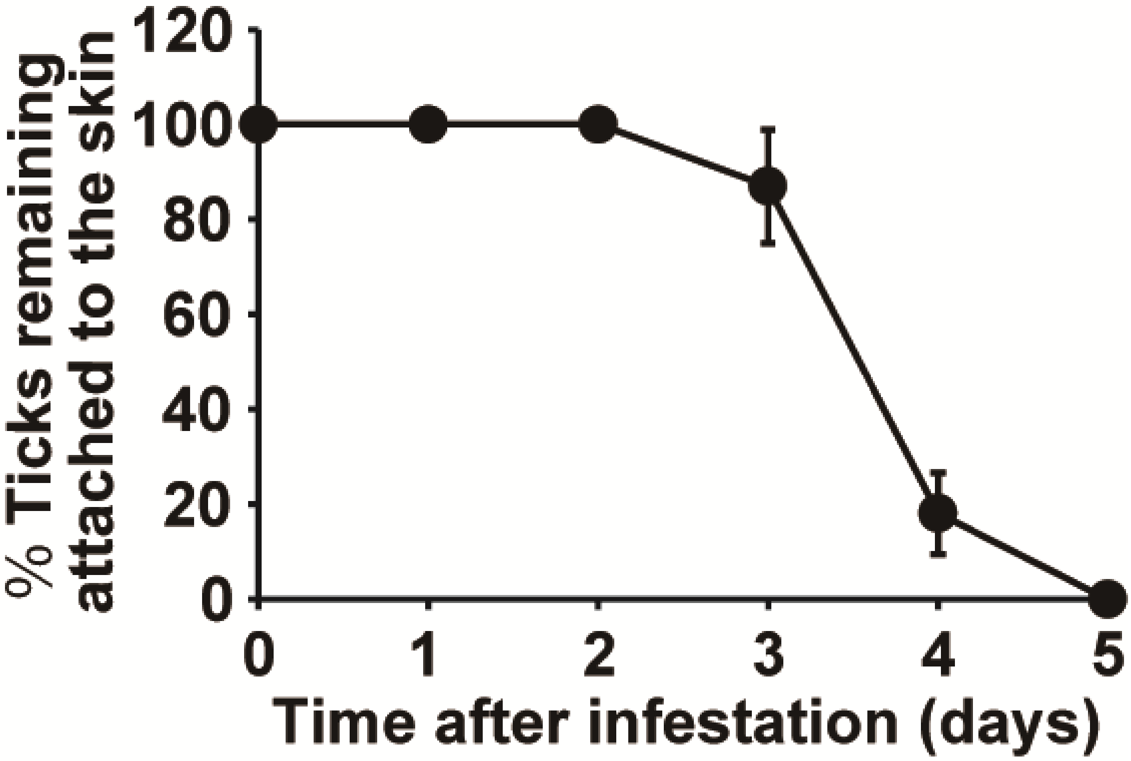

Fig. S1. Time course of the number of ticks staying attached to the skin during the 2nd infestation

C57BL/6 mice were infested twice with ticks as in Fig. 1A, and the number of ticks staying attached to the skin (mean ± SEM, n=4 each) was counted at the indicated time points. The proportion (%) of skin-attached ticks at each point was calculated and displayed where the number of skin-attached ticks on day 0 of infestation is set as 100%. Data shown are representative of 2 independent experiments.
