# Supplementary material for: Skin CD4+ Memory T Cells Play an Essential Role in Acquired Anti-Tick Immunity through Interleukin-3-Mediated Basophil Recruitment to Tick-Feeding Sites
Source: Front Immunol. 2017 Oct 16;8:1348. doi: 10.3389/fimmu.2017.01348 (PMC5650685; doi:10.3389/fimmu.2017.01348)
Supplement: Supplementary file 3 [file image_2.pdf]

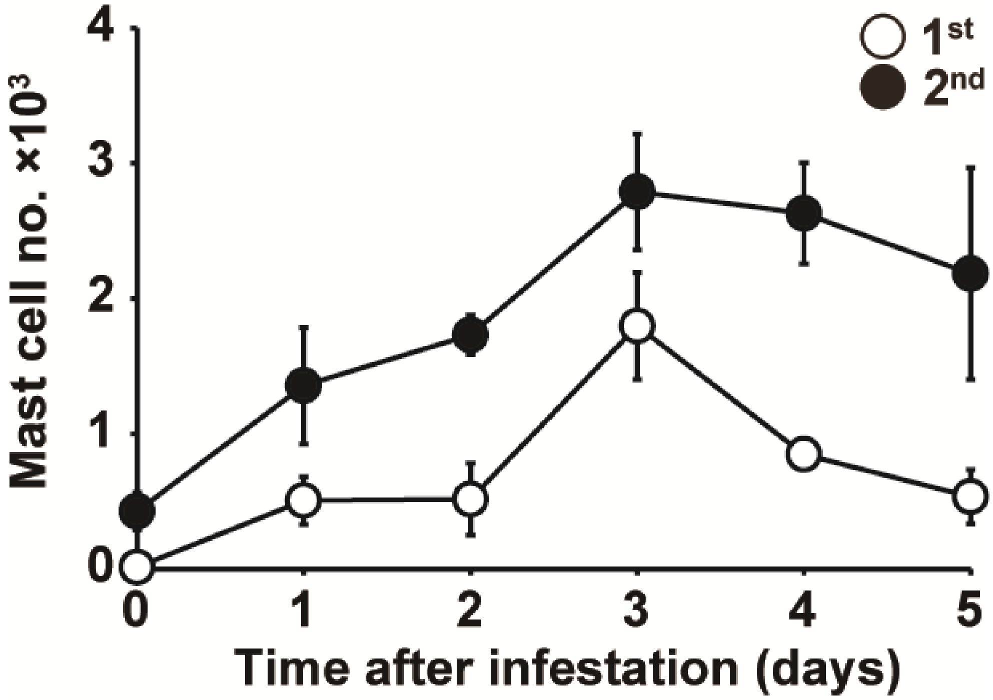

Fig. S2. Time course of the number of mast cells at tick-feeding sites during infestation

C57BL/6 mice were infested once or twice with ticks as in Fig. 1A. The number of mast cells at tick-feeding sites (mean ± SEM, n=3 each) was counted at the indicated time points during the 1st (white circles) and 2nd (black circles) infestations. Data shown are representative of 2 independent experiments.
