# Supplementary material for: Skin CD4+ Memory T Cells Play an Essential Role in Acquired Anti-Tick Immunity through Interleukin-3-Mediated Basophil Recruitment to Tick-Feeding Sites
Source: Front Immunol. 2017 Oct 16;8:1348. doi: 10.3389/fimmu.2017.01348 (PMC5650685; doi:10.3389/fimmu.2017.01348)
Supplement: Supplementary file 4 [file image_3.pdf]

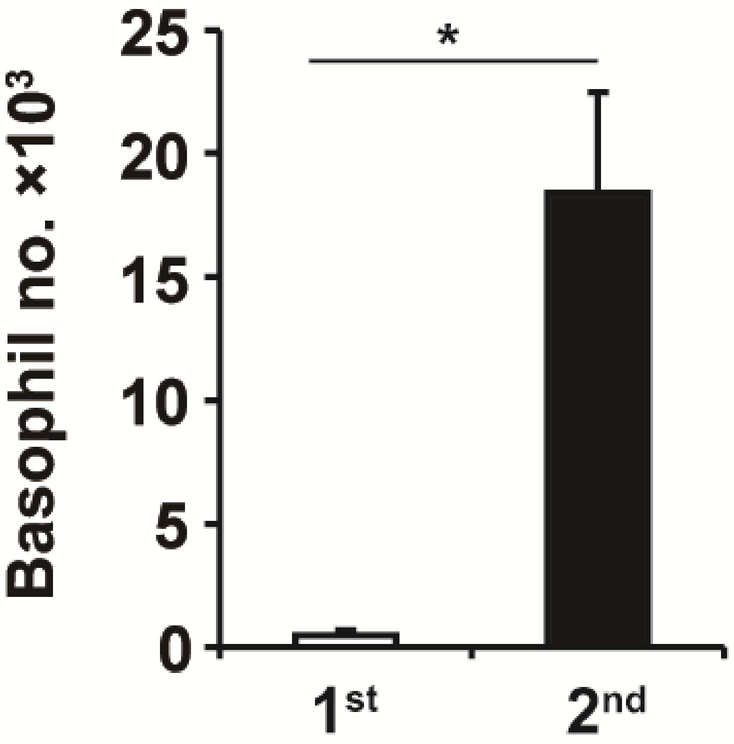

Fig. S3. No significant accumulation of basophils at the 1st tick-feeding site in *Rag2*$^{-/-}$ mice into which CD4^{+} T cells from WT mice were adoptively transferred

CD4^{+} T cells from WT mice were adoptively transferred to *Rag2*$^{-/-}$ mice as shown in Fig. 2C. Recipient mice were infested once or twice with ticks, and the number of basophils at tick-feeding site was examined on day 2 of infestation (mean ± SEM, n=4 each). Data shown are representative of 2 independent experiments. *$P<0.05$.
