# Supplementary material for: Skin CD4+ Memory T Cells Play an Essential Role in Acquired Anti-Tick Immunity through Interleukin-3-Mediated Basophil Recruitment to Tick-Feeding Sites
Source: Front Immunol. 2017 Oct 16;8:1348. doi: 10.3389/fimmu.2017.01348 (PMC5650685; doi:10.3389/fimmu.2017.01348)
Supplement: Supplementary file 5 [file image_4.pdf]

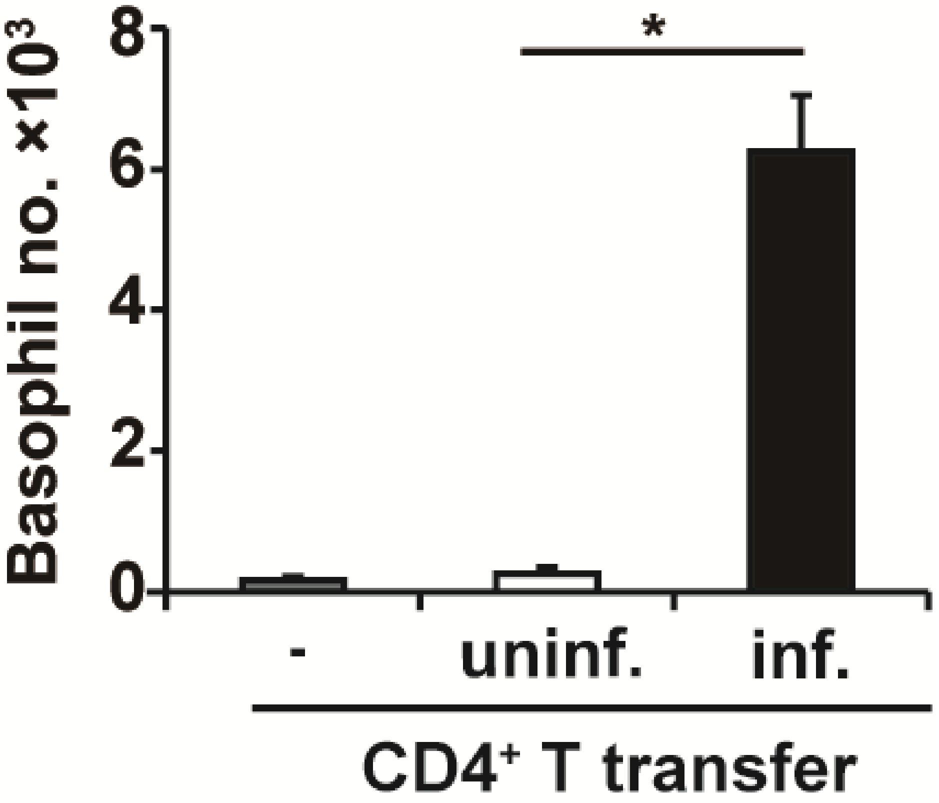

Fig. S4. Adoptive transfer of $CD4^+$ T cells isolated from previously tick-infested mice confers basophil accumulation at the tick-feeding site even in the 1st infestation on *Rag2*$^{-/-}$ mice

$CD4^+$ T cells were isolated from uninfested mice or mice infested once with ticks 14 days before, and adoptively transferred to *Rag2*$^{-/-}$ mice. Recipient mice were infested once with ticks 7 days later, and the number of basophils (mean ± SEM, n=4 each) at the tick-feeding site was examined on day 2 of infestation. As a control, *Rag2*$^{-/-}$ mice without T cell transfer were similarly infested and analyzed. Data shown are representative of 2 independent experiments. *P<0.05.
