# Supplementary material for: Skin CD4+ Memory T Cells Play an Essential Role in Acquired Anti-Tick Immunity through Interleukin-3-Mediated Basophil Recruitment to Tick-Feeding Sites
Source: Front Immunol. 2017 Oct 16;8:1348. doi: 10.3389/fimmu.2017.01348 (PMC5650685; doi:10.3389/fimmu.2017.01348)
Supplement: Supplementary file 6 [file image_5.pdf]

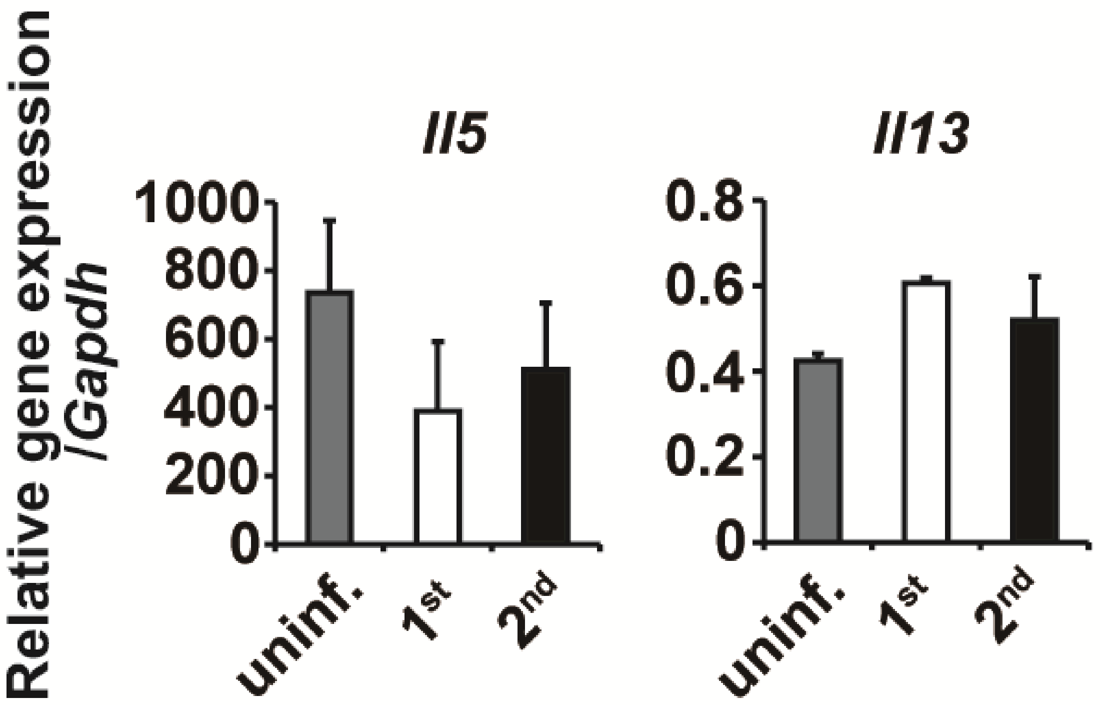

Fig. S5. No significant upregulation of *Il5* and *Il13* expression at the tick-feeding site

The transcriptional expression of *Il5* and *Il13* at uninfested skin, 1st or 2nd tick-feeding site on day 2 of infestation is shown (mean ± SEM, n=3 each). Data shown are representative of 2 independent experiments.
