# Supplementary material for: Skin CD4+ Memory T Cells Play an Essential Role in Acquired Anti-Tick Immunity through Interleukin-3-Mediated Basophil Recruitment to Tick-Feeding Sites
Source: Front Immunol. 2017 Oct 16;8:1348. doi: 10.3389/fimmu.2017.01348 (PMC5650685; doi:10.3389/fimmu.2017.01348)
Supplement: Supplementary file 7 [file image_6.pdf]

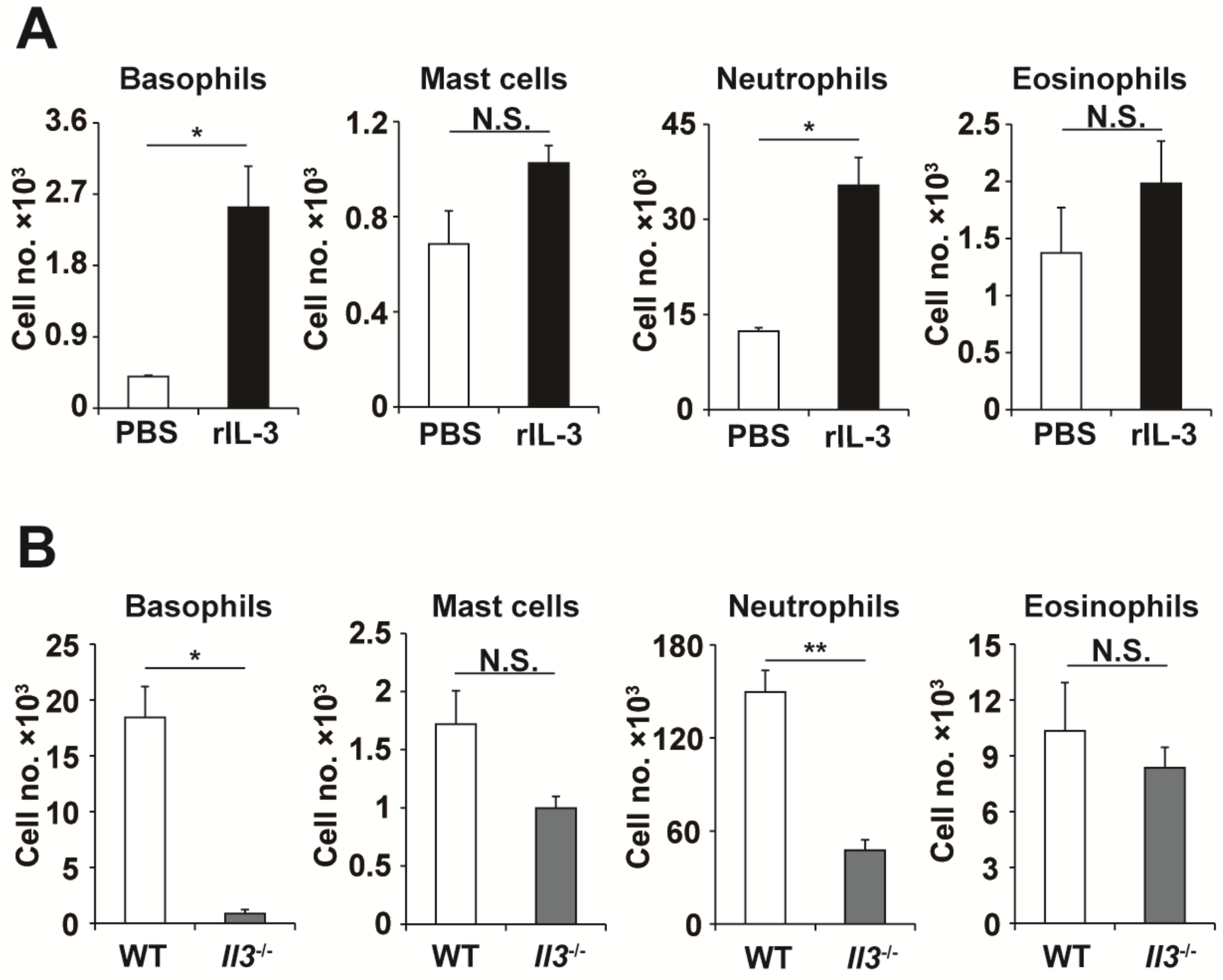

Fig. S6. CD4$^+$ T cell-derived IL-3 contributes to the accumulation of basophils and neutrophils at the 2nd tick-feeding sites

(A) *Rag2*$^{-/-}$ mice were treated with rIL-3 or control PBS and infested once with ticks as shown in Fig. 3D. The numbers of basophils, mast cells, neutrophils and eosinophils (mean ± SEM, n=4 each) at the 1st tick-feeding site of each side of flank were examined on day 2 of infestation. (B) CD4$^+$ T cells from WT or *Il3*$^{-/-}$ mice were adoptively transferred into *Rag2*$^{-/-}$ mice, and recipient mice were infested twice with ticks as shown in Fig. 4A. The number of basophils, mast cells, neutrophils and eosinophils at the 2nd tick-feeding site was examined on day 2 of infestation (mean ± SEM, n=4 each). Data shown are representative of 2 independent experiments. N.S., not significant; *P<0.05; **P<0.01.
