# Supplementary material for: Skin CD4+ Memory T Cells Play an Essential Role in Acquired Anti-Tick Immunity through Interleukin-3-Mediated Basophil Recruitment to Tick-Feeding Sites
Source: Front Immunol. 2017 Oct 16;8:1348. doi: 10.3389/fimmu.2017.01348 (PMC5650685; doi:10.3389/fimmu.2017.01348)
Supplement: Supplementary file 8 [file image_7.pdf]

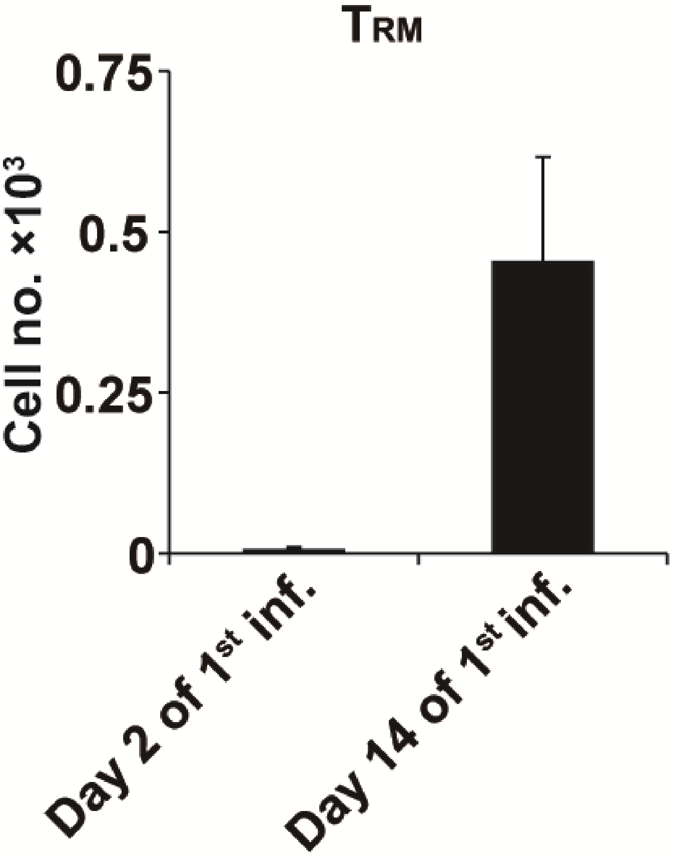

Fig. S7. CD4$^+$ T$_{RM}$ cells can be detected at previously-uninfested skin on day 14 of the 1st infestation in *Rag2$^{-/-}$* mice that received CD4$^+$ T cells from WT mice

CD4$^+$ T cells from WT mice were adoptively transferred into *Rag2$^{-/-}$* mice, and recipient mice were infested once with ticks. On day 2 or day 14 of infestation, previously-uninfested skin was isolated from the opposite flank to examine the number of CD4$^+$ T$_{RM}$ cells (mean ± SEM, n=3 each). Data shown are representative of 2 independent experiments.
